# The Role of Lymphadenectomy in Surgical Staging of Endometrial Cancer

**DOI:** 10.1155/2011/814649

**Published:** 2011-07-10

**Authors:** Nikki L. Neubauer, John R. Lurain

**Affiliations:** ^1^Division of Gynecologic Oncology, Robert H. Lurie Comprehensive Cancer Center, Northwestern University Feinberg School of Medicine, Chicago, IL, USA; ^2^Prentice Women's Hospital, 250 E. Superior St., Suite 05-2168, Chicago, IL 60611, USA

## Abstract

Surgical staging, including lymph node sampling, for endometrial cancer was adopted by the International Federation of Gynecology and Obstetrics (FIGO) in 1988 based on reports demonstrating diagnostic and therapeutic advantages. This review focuses on the incidence of lymph node metastasis, risk factors for lymph node involvement, the effect of lymph node metastasis on prognosis, the therapeutic effect and diagnostic usefulness of lymphadenectomy, risks of lymph node dissection, and future directions in surgical staging of endometrial cancer. Surgical staging identifies most patients with extrauterine disease as well as uterine risk factors for recurrence, thereby allowing for a more informed approach to postoperative adjuvant therapy. Lymphadenectomy as a part of surgical staging is not required in patients assessed intraoperatively to be at low risk for lymph node metastasis (<2 cm grade 1 tumors with superficial myometrial invasion), however, a systematic lymph node dissection should be performed in most other patients with endometrial cancer. In the future, molecular markers may be useful to predict preoperatively tumor aggressiveness and lymph node metastasis. It is hoped that an approach of surgical staging with selective lymph node dissection will improve survival and spare patients additional surgical complications or unnecessary postoperative exposure to radiation and/or chemotherapy.

## 1. Introduction

Endometrial cancer is the most common gynecologic malignancy in the United States with an estimated 43,470 new cases and 7,950 cancer-related deaths in 2010 [1]. Most patients are diagnosed with uterine-confined disease, which can usually be cured by surgery alone. The presence of extrauterine disease significantly affects recurrence rates and survival, which emphasizes the importance of identification of sites of disease spread and provision of appropriate adjuvant postoperative therapy. 

In 1987, the Gynecologic Oncology Group (GOG) published results of Protocol 33, which investigated the usefulness of surgical staging in patients with endometrial cancer clinically confined to the uterus. This study found that 22% of patients had disease spread outside the uterus, including disease spread to pelvic lymph nodes in 9% and para-aortic lymph nodes in 5% [2]. In response to this paper and others, the International Federation of Gynecology and Obstetrics (FIGO) changed to a surgical staging system for endometrial cancer in 1988 [3]. 

Since the establishment of surgical staging as the standard initial step in the management of most patients with endometrial cancer, the performance of pelvic and para-aortic lymphadenectomy as part of surgical staging has increased due to reports showing diagnostic and therapeutic advantages [4, 5]. Subsequently, in 2006, the American Congress of Obstetricians and Gynecologists (ACOG) recommended surgical staging, including lymph node sampling, for most women with endometrial cancer except young women desiring fertility preservation and women at increased risk of mortality secondary to comorbidities [6]. Despite these recommendations, lymph node dissection as a part of endometrial cancer staging, especially in “low risk” patients, is still controversial and staging practices vary widely based upon individual physician and institutional practices. In a recent survey of members of the Society of Gynecologic Oncologists, 35%, 66%, and 90% of respondents routinely performed a lymph node dissection as part of endometrial cancer staging in grade 1, 2, and 3 tumors, respectively. In addition, most members (53% of respondents) did not use intraoperative frozen section to guide decisions about lymph node dissection [7]. 

This paper on lymph node dissection as a component of surgical staging of endometrial cancer will focus on the incidence of lymph node metastasis, risk factors for lymph node involvement, the effect of lymph node metastasis on prognosis, the therapeutic effect of lymph node dissection, and the diagnostic usefulness of lymph node dissection in directing postoperative adjuvant therapy. 

## 2. Risk Factors for Lymph Node Metastases

Tumor grade and depth of myometrial invasion appear to be the two most important factors in determining the risk for lymph node metastasis. In GOG Protocol 33, Creasman et al. reported that the overall incidence of lymph node metastasis in clinically uterine-confined endometrial cancer was about 3% in grade 1, 9% in grade 2, and 18% in grade 3 tumors. Less than 5% of patients with <50% myometrial invasion had lymph node metastasis compared with about 20% of patients with >50% myometrial invasion. Pelvic lymph node metastases were present in less than 5% of grade 1 and 2 tumors with <50% myometrial invasion, in about 15% of grade 1 and 2 tumors with >50% myometrial invasion or grade 3 tumors with <50% myometrial invasion, and in more than 40% of grade 3 tumors with >50% myometrial invasion [2]. Boronow et al. noted that patients with outer one-third myometrial invasion had a 25% incidence of pelvic lymph node metastasis and a 17% incidence of para-aortic lymph node metastasis compared to only a 1% incidence of a lymph node metastasis in patients without myometrial invasion [8]. Chi et al. reporting on the incidence of lymph node metastasis in patients with surgically staged endometrioid endometrial cancer confirmed that as tumor grade increased the risk of myometrial invasion also increased [9]. In their series, no patient with grade 1 tumor on final pathology and only 2% of patients with no myometrial invasion had lymph node metastasis.

The incidence of lymph node metastasis also correlates with tumor size (<2 cm, 4%; >2 cm, 15%; entire cavity, 35%) [10, 11]. Schink et al. from Northwestern and Mariani et al. from the Mayo Clinic reported that no patient with endometrial grade 1 or 2 tumors and <50% myometrial invasion had lymph node metastasis when the tumor size was ≤2 cm [10, 11]. Cervical involvement is associated with about a 15% risk of lymph node metastasis [12]. Extrauterine spread of diseases increases the risk of lymph node metastasis. Adnexal metastasis increases the risk for pelvic and para-aortic nodal metastasis to 32% and 20%, respectively [12]. Positive peritoneal cytology has been found to be associated with deep myometrial invasion, cervical involvement, adnexal spread, and lymph node metastasis [13]. In a prospective evaluation of peritoneal cytology in patients with clinical stage I endometrial cancer who underwent primary surgical therapy at Northwestern, we found that patients with malignant pelvic washings often had other poor prognostic factors, including deep myometrial invasion in 37%, grade 3 tumors in 37%, and positive lymph nodes in 17% [14].

## 3. Prognostic Importance of Lymph Node Metastasis

Lymph node metastasis is the most important prognostic factor in clinical stage 1 endometrial cancer. Of patients with clinical stage I disease, about 10% will have pelvic and 6% will have para-aortic lymph node metastases [15]. Patients with lymph node metastases have an almost sixfold higher likelihood of developing recurrent cancer compared to patients without lymph node metastases. Morrow et al. evaluated the relationship between surgical-pathologic risk factors and outcomes in clinical stage I and II endometrial cancer using the GOG 33 database [15]. They noted that the five-year recurrence-free survival was 90% in patients without lymph node metastases, 75% in patients with pelvic lymph node metastases, and 38% in patients with positive para-aortic lymph nodes. In fact, the strongest predictor of recurrence was lymph node status. Lurain et al. subsequently confirmed an overall recurrence rate of 48% with positive lymph nodes, including 45% with positive pelvic nodes and 64% with positive para-aortic nodes, compared to 8% with negative nodes. The five-year disease-free survival rate for patients with lymph node metastases was 54% compared with 90% for patients without lymph node metastases [16]. Mariani et al. noted that just the presence or absence of para-aortic lymph node metastases was of paramount importance. Of para-aortic node-positive patients, 58% developed progressive or recurrent cancer, and 90% of patients with a para-aortic recurrence died of disease [17]. 

Mariani et al. evaluated patients with lymph node metastases in addition to other extrauterine sites of disease (vagina, uterine serosa, positive peritoneal cytology, and adnexal involvement). The recurrence rate was 32% (5% extranodal) for those with lymphatic dissemination only compared to 67% (41% extranodal) for those with additional sites of extrauterine disease spread [18]. Similarly, Lurain et al. noted that patients with lymph node metastases who also had involvement of the uterine serosa, positive peritoneal cytology, and/or adnexal metastasis had a two-year overall survival of 50% compared to 88% in patients with only nodal extrauterine disease [16].

At Northwestern, we evaluated the clinicopathologic characteristics and survival of patients with pelvic and para-aortic lymph node metastases [19]. Five-year disease-specific survival was 72%, and five-year overall survival was 61%, including 70% for those with pelvic node metastases only and 49% for those with para-aortic metastases. On multivariate analysis, advancing patient age, nonendometrioid histology, and ≥50% myometrial invasion were significantly associated with poorer overall survival. Disease recurred in 25% of patients, and all recurrences were outside of the adjuvant radiation field. 

## 4. Therapeutic Value of Lymph Node Dissection

Several retrospective studies have lent credence to the idea that surgical staging with lymph node dissection can not only assist with directing postoperative adjuvant therapy, but may even have a therapeutic effect. Havrilesky et al. in 2005 found that in a group of patients with pelvic or para-aortic metastases, disease-specific survival was improved if microscopic versus gross disease was removed [20]. This group also noted that gross nodal disease not debulked was a significant poor prognostic factor in these patients. In patients with IIIC endometrial cancer, lymph node involvement is a poor prognostic factor, and there appears to be a therapeutic benefit to lymph node dissection and resection in these patients. In a group of patients with FIGO stage I and II endometrial cancer, Lutman et al. found that pelvic lymph node count was an important prognostic factor in patients with grade 3 tumors and clear cell or papillary serous histology [21]. The authors also stratified patients by risk factors including myometrial invasion, histology, and age, and they found that a pelvic lymph node count of ≥12 was an important prognostic factor for survival in patients of high-intermediate risk (age >70 with 1 risk factor or <70 with 2 risk factors). From this paper, it appears that a pelvic lymph node count ≥12 was associated with improved survival in patients of high-intermediate risk and in patients with stage I and II endometrial cancer with high-risk histology. 

In a comparison of patients with endometrial cancer in the SEER database, Chan et al. found that in the intermediate/high-risk patients (Stage IB, grade 3; Stage IC and II-IV, all grades), lymph node dissection was associated with improved 5-year disease-specific survival. Extensive nodal resection also improved disease-specific survival in stage IIIC-IV patients. There was no survival benefit seen in patients with low-risk disease [22]. Chan et al. also studied data from the National Cancer Institute database on close to 40,000 women with endometrioid endometrial cancer [23]. Comparing women who underwent surgical staging with lymph node dissection with those who did not have a lymph node dissection, they noted 5-year disease-specific survival for women with disease stages I, II, III, IV who underwent lymphadenectomy of 96%, 90%, 74%, and 53% as compared to women not undergoing lymphadenectomy of 97%, 82%, 63%, and 27%, respectively for each stage. No survival benefit was noted for women with stage I, grade 1 disease. Similarly, Neubauer et al. found no survival benefit to lymph node dissection in patients with preoperative grade 1 endometrial cancer [24]. They also noted that pelvic and para-aortic node metastases were identified in 5.4% and 3.2%, respectively, of patients undergoing lymphadenectomy and that 25% of patients were upgraded on final pathology, including 22% upgraded to grade 2 and 3% upgraded to grade 3. 

Three other retrospective studies have suggested a therapeutic benefit to lymphadenectomy. Kilgore et al. evaluated survival in 649 patients who underwent surgery for clinical stage I or II endometrial adenocarcinoma. After three years of mean followup, patients undergoing multiple-site pelvic lymph node dissection had improved overall 5-year survival as compared to patients who did not undergo lymph node dissection, 90% versus 70% [25]. In patients at high risk of para-aortic node metastases (myometrial invasion >50%, positive pelvic lymph nodes, or adnexal involvement), Mariani et al. noted an improved survival in patients undergoing para-aortic lymphadenectomy as compared to those who did not undergo para-aortic lymph node dissection [17]. The 5-year progression-free survival was 36% in those who did not undergo staging with para-aortic lymph node dissection versus 76% in those undergoing para-aortic lymph node dissection; the overall survival was improved as well (42% versus 77%). Similarly, Todo et al. noted that overall survival was longer in patients with endometrial cancer undergoing pelvic and paraaortic lymphadenectomy versus pelvic lymphadenectomy alone (HR 0.53) [26]. In patients at intermediate to high risk of recurrence (IA grade 3, IB and IC any grade, stage II–IV disease), a more pronounced effect was noted for pelvic and para-aortic lymphadenectomy with longer overall, disease-specific, and recurrence-free survival noted in the group of these patients undergoing both pelvic and para-aortic lymphadenectomy as compared to pelvic lymphadenectomies alone. A 10.6% increase in 5-year overall survival was noted in intermediate to high-risk patients undergoing both pelvic and para-aortic lymphadenectomies.

 Lymph node dissection may also assist with postoperative treatment planning in patients with endometrial cancer. In a retrospective review of 349 patients undergoing surgical management for endometrial cancer, Ben-Shachar et al. discovered that 12% of patients received adjuvant therapy postoperatively but that based upon the results of surgical staging 17% were able to avoid adjuvant therapy [27]. Thus, in an otherwise high-risk patient, the use of adjuvant radiation or chemotherapy may be averted based upon the results of surgical staging including lymph node dissection. Selvaggi et al. also found that surgical staging identified patients with positive lymph nodes who would not have otherwise received postoperative radiation, but they also found that the pattern and location of recurrences was not different between patients undergoing and not undergoing lymphadenectomy [28]. 

The question remains as to whether lymph node dissection confers a survival benefit to patients. A large number of patients would be required to reliably answer this question, as most patients with endometrial cancer survive more than five years and often die of comorbidities first. Bernardini et al. performed another retrospective review comparing the surgical practices of two academic centers with regard to surgical staging of grade 1 endometrial cancer [29]. 494 women were included in the study and were similar in terms of grade, final histology, and type of hysterectomy. Patients in the institution that did not routinely perform surgical staging with lymph node dissection were on average older, more likely to have lymphovascular space invasion, and cervical involvement. Lymphadenectomy was performed in 49.4% versus 11.7% of patients in the institution that did not routinely perform surgical staging. Overall survival was similar at both institutions (96% versus 96%), and three-year recurrence-free survival was also similar. Thus in low-risk endometrial cancer, there may be no difference in recurrence-free or overall survival within the first five years of surgical management.

## 5. Prospective Data regarding Therapeutic Benefit of Lymph Node Dissection in Endometrial Cancer

Two prospective randomized trials have recently been published which shed more light onto the topic of lymph node dissection, in endometrial cancer. The ASTEC study was a randomized controlled trial involving 85 institutions in 4 countries [30]. 1408 patients were randomized to abdominal hysterectomy, bilateral salpingo-oophorectomy with or without pelvic lymph node dissection and then subsequently to the use of postoperative radiation. The study was designed to detect an improvement in overall 5-year survival from 80% in the standard arm to 90% in the patients undergoing lymphadenectomy. There was no difference in overall survival between the two treatment groups. The study has been criticized due to noncompliance with regard to lymph node dissection as well as the small average number of lymph nodes removed. In addition, some patients were randomized to postoperative radiation irrespective of surgical pathology, which may have skewed survival data.

Panici et al. reported on a phase 3 randomized trial comparing standard surgery of total hysterectomy with bilateral salpingo-oophorectomy with or without planned lymph node dissection for early-stage endometrial carcinoma [31]. In this study, the median number of lymph nodes removed was twenty-eight and patients were comparable in their characteristics across both arms. A greater number of lymph node metastases was detected in the lymphadenectomy arm (13.3% versus 3.2%), and early and late postoperative complications occurred statistically more frequently in the lymphadenectomy arm. Similar proportions of women in both groups received adjuvant therapy (68.9% versus 64.8%). Of patients developing recurrent cancer, median time to relapse was similar in both groups (14 months versus 13 months), and overall 5-year survival was not significantly different (85.9% in the lymph node dissection arm versus 90% in the standard surgery alone arm). This study did not have standard criteria for the use of postoperative adjuvant therapy and did not consider recurrence risk in the analysis of survival. 

Both of these studies found that lymphadenectomy did not confer a survival advantage in a low-risk group of endometrial cancer patients, but that lymphadenectomy did allow for more accurate staging with a significant number of patients upstaged in the Panici et al. study. Both studies were limited in that postoperative adjuvant therapy was not controlled and pelvic lymphadenectomy alone was performed instead of a systematic pelvic and para-aortic lymphadenectomy. Both studies came to similar conclusions, supporting previously gathered retrospective data, that patients with endometrial cancer and low-risk factors for recurrence may not require a systematic lymphadenectomy in contrast to patients with intermediate or high-risk factors for recurrence.

## 6. Long-Term Risks of Lymph Node Dissection

A longstanding argument against routine pelvic lymph node dissection is that the procedure can involve risks for the patient including vascular injury intraoperatively, but more importantly, long-term risks such as lymphedema and lymphocyst. In an analysis of the surgical complications associated with surgical staging in 128 patients, Orr et al. determined the long-term risk of lymphocyst to be 1.3% and lymphedema to be 0.7%, both acceptable risks for a surgical procedure which can assist a physician with the quantification of a patient's risk for recurrence [32]. Recently, Todo et al. performed a retrospective chart review evaluating the risk factors associated with postoperative lower extremity lymphedema and found that 38% of patients undergoing exploratory laparotomy, total abdominal hysterectomy, bilateral salpingo-oophorectomy, and pelvic and para-aortic lymph node dissection experienced postoperative lower extremity lymphedema, a much higher percentage than cited in other studies. The authors found that adjuvant pelvic external beam radiation therapy, resection of more than thirty-one lymph nodes, and removal of circumflex iliac nodes were risk factors for the development of lower extremity lymphedema [33]. 

As more patients undergo minimally invasive surgery for the treatment of endometrial cancer there is the question of whether or not laparoscopic surgery can decrease the risk of postoperative complications including lymphedema and lymphocele. Kong et al. also compared the surgical outcomes of laparoscopic surgery and open surgery in patients with endometrial cancer [34]. Patients undergoing laparoscopy had on average 4 more lymph nodes removed and 11.7% of these patients were diagnosed with a lymphocyst while 10% of patients undergoing laparotomy were diagnosed with a lymphocyst. Again, the risk of lymphedema and lymphocystes appears to be related to the number of lymph nodes removed. The percentage of patients experiencing lymphedema varies within the literature and has increased in prevalence, but this may simply be due to greater attention to this postoperative complication. 

Several studies have verified the minimally invasive approach to the surgical treatment of endometrial cancer and support the notion that laparoscopy may benefit patients, especially those at high risk for postoperative complications [35, 36]. Two prospective randomized trials have also proven the safety of laparoscopy in the treatment of endometrial cancer [36, 37]. The Gynecologic Oncology Group LAP2 study compared surgical complications and short-term outcomes in 2616 patients with clinical stage I-IIA endometrial cancer randomly assigned to laparotomy (920) or laparoscopy (1696) [37]. All patients were to undergo standardized surgical staging. Intraoperative complications were similar between the two groups (8% for laparotomy and 10% for laparoscopy), while postoperative complications were more common in laparotomy patients (21% versus 14%). Pelvic lymph node dissection was performed in 99% of laparotomy patients and 98% of laparoscopy patients. No prospective studies exist which directly compare postoperative lymphedema and lymphocyst rates as a primary endpoint in patients undergoing lymphadenectomy for endometrial cancer; however, LAP2 proved that a minimally invasive approach to surgical staging of endometrial cancer is a reasonable alternative to the standard exploratory laparotomy [37], while the trial by Tozzi et al. proved that survival of patients with endometrial cancer undergoing minimally invasive surgery is comparable to those patients undergoing treatment for endometrial cancer via laparotomy [36]. 

## 7. Future Directions in Surgical Staging of Endometrial Cancer

In light of the above survival findings of both retrospective and prospective trials, it seems that a group of low-risk patients exist who may not need routine lymph node dissection. Mariani et al. at the Mayo Clinic published an algorithm for performing lymph node dissection in patients with endometrial cancer [38]. Patients not requiring lymph node dissection are those with a 2 cm or smaller tumor, grade 1 or 2 disease, endometrioid histology, myometrial invasion of 50% or less, and no evidence of gross metastatic disease. The authors found that by using this algorithm, no patient in the low-risk group had nodal metastasis. This model may only work, however, in select centers that have access to a dedicated gynecologic pathologist who can provide accurate intraoperative assessment thereby decreasing the chance of significant upstaging. Lee et al. constructed a preoperative prediction model to help determine which patients can avoid lymph node dissection in order to bypass the need for a pathologist at the time of intraoperative assessment [39]. Eligible patients were those who underwent surgical staging and lymph node dissection for endometrial cancer. Preoperative predictors comprising the scoring system included CA-125, MRI to assess myometrial invasion, histologic grade, and clinical stage. Patients in the low-risk group (score of 0-1) had no nodal metastases, whereas patients in the high-risk group (score 3-4) had a frequency of nodal metastasis of 50%. These studies argue for the triage of endometrial cancer patients into low-risk and high-risk groups, thereby sparing low-risk patients from undergoing a full lymph node dissection. 

Another future development in surgical staging of endometrial cancer may lie in sentinel lymph node detection. Several studies have validated the use of sentinel lymph node detection in experienced hands in other gynecologic cancers. Similarly in endometrial cancer, authors have noted that the sentinel node detection rate ranges from 77% to 94% depending on provider experience level and that the false negative rate decreases with increasing provider experience [40]. Khoury-Collado et al. evaluated the sentinel lymph node detection rate after cervical injection of blue dye and found that the detection rate at the beginning of the study was much lower than at the end of the study (78% versus 94%) [41]. Several methods of injection of dye have been evaluated and none have proven superior thus far. Mais et al. compared the sentinel node detection rate in patients with clinical stage I-II endometrial cancer randomized to either exploratory laparotomy or laparoscopically assisted vaginal hysterectomy [42]. The authors found that the sentinel lymph node detection rate was higher in laparoscopic procedures (82%) versus laparotomy (41%), but that overall only 50% of patients with lymph node metastases were detected. Sentinel lymph node detection cannot as of yet be considered equal to a complete lymphadenectomy in endometrial cancer. Larger studies are needed to determine the best method of dye injection, the optimal patient this procedure will benefit, and the efficacy of sentinel lymph node detection in endometrial cancer.

In the near future, the oncology community may also use molecular markers to predict the metastatic potential of endometrial cancer. Mutations in the PTEN gene causing loss of function have been reported in 25–83% of endometrioid carcinomas. P53 mutations have also been reported in endometrial carcinomas, more often in papillary serous histology. Overexpression of P53, altered P16 expression, and high Ki-67 expression have all been associated with a poor prognosis, while mutations or deletions of the PTEN gene tend to be associated with well-differentiated and less invasive tumors [43]. The DNA ploidy index is another molecular prognostic factor which may predict the aggressiveness of an endometrial cancer [44]. Type 2 endometrial carcinomas are more likely to be nondiploid, while endometrioid carcinomas are more likely to be diploid. DNA ploidy may prove useful preoperatively to help determine which patients are at higher risk of lymph node metastasis and postoperatively to determine which patients are more likely to need adjuvant therapy. 

In an attempt to further determine preoperatively which patients with endometrial cancer are more likely to need a lymphadenectomy, Trovik et al. examined stathmin expression which is tied to the AKT signaling pathway [45]. Stathmin expression was correlated with non-endometrioid histology, aneuploidy, and higher grade. Stathmin expression was also found to be an independent predictor of lymph node metastases. Clearly, molecular markers are useful at this time to predict tumor aggressiveness and nonendometrioid histology but further studies are needed to find a marker that can predict lymph node metastases in grade 1 or 2 endometrioid carcinomas. 

## 8. Conclusion

After review of the literature, we conclude that surgical staging not only identifies most patients with extrauterine disease, but also identifies patients with uterine risk factors for recurrence, including large tumor size, deep myometrial invasion, LVSI, cervical extension, and positive peritoneal cytology. Identification of these extrauterine and intrauterine risk factors allows for a more informed approach to the use of postoperative adjuvant therapy. A systematic lymph node dissection should be performed in patients with endometrial cancer who are at intermediate to high risk of lymph node metastases. Lymph node biopsies are not required in patients assessed intraoperatively to be at very low risk for lymphatic metastasis (i.e., patients with small (<2 cm) grade 1 endometrial cancers with only superficial myometrial invasion). There are documented risks associated with lymphadenectomy, but these risks decrease in the hands of an experienced surgeon. It is hoped that this approach of surgical staging followed by postoperative targeted treatment will improve survival and spare patients unnecessary exposure to radiation and/or chemotherapy.

## References

[1] http://www.cancer.gov/.

[2] Creasman WT, Morrow CP, Bundy BN, Homesley HD, Graham JE, Heller PB (1987). Surgical pathologic spread patterns of endometrial cancer. A gynecologic oncology group study. *Cancer*.

[3] International Federation of Gynecology and Obstetrics (1989). Annual report on the results of treatment in gynecologic cancer. *International Journal of Gynecology & Obstetrics*.

[4] Kilgore LC, Partridge EE, Alvarez RD (1995). Adenocarcinoma of the endometrium: survival comparisons of patients with and without pelvic lymph node sampling. *Gynecologic Oncology*.

[5] Mariani A, Webb MJ, Galli L, Podratz KC (2000). Potential therapeutic role of para-aortic lymphadenectomy in node-positive endometrial cancer. *Gynecologic Oncology*.

[6] ACOG Committee on Practice Bulletins (2005). Management of endometrial cancer.

[7] Soliman PT, Frumovitz M, Spannuth W (2010). Lymphadenectomy during endometrial cancer staging: practice patterns among gynecologic oncologists. *Gynecologic Oncology*.

[8] Boronow RC, Morrow CP, Creasman WT (1984). Surgical staging in endometrial cancer: clinical-pathologic findings of a prospective study. *Obstetrics and Gynecology*.

[9] Chi DS, Barakat RR, Palayekar MJ (2008). The incidence of pelvic lymph node metastasis by FIGO staging for patients with adequately surgically staged endometrial adenocarcinoma of endometrioid histology. *International Journal of Gynecological Cancer*.

[10] Schink JC, Lurain JR, Wallemark CB, Chmiel JS (1987). Tumor size in endometrial cancer: a prognostic factor for lymph node metastasis. *Obstetrics and Gynecology*.

[11] Mariani A, Webb MJ, Keeney GL, Lesnick TG, Podratz KC (2002). Surgical stage I endometrial cancer: predictors of distant failure and death. *Gynecologic Oncology*.

[12] Disaia PJ, Creasman WT, Boronow RC, Blessing JA (1985). Risk factors and recurrent patterns in stage I endometrial cancer. *American Journal of Obstetrics and Gynecology*.

[13] Creasman WT, Disaia PJ, Blessing J, Wilkinson RH, Johnston W, Weed JC (1981). Prognostic significance of peritoneal cytology in patients with endometrial cancer and preliminary data concerning therapy with intraperitoneal radiopharmaceuticals. *American Journal of Obstetrics and Gynecology*.

[14] Lurain JR, Rumsey NK, Schink JC, Wallemark CB, Chmiel JS (1989). Prognostic significance of positive peritoneal cytology in clinical stage I adenocarcinoma of the endometrium. *Obstetrics and Gynecology*.

[15] Morrow CP, Bundy BN, Kurman RJ (1991). Relationship between surgical-pathological risk factors and outcome in clinical stage I and II carcinoma of the endometrium: a Gynecologic Oncology Group study. *Gynecologic Oncology*.

[16] Lurain JR, Rice BL, Rademaker AW (1991). Prognostic factors associated with recurrence in clinical stage I adenocarcinoma of the endometrium. *Obstetrics & Gynecology*.

[17] Mariani A, Webb MJ, Galli L, Podratz KC (2000). Potential therapeutic role of para-aortic lymphadenectomy in node-positive endometrial cancer. *Gynecologic Oncology*.

[18] Mariani A, Webb MJ, Keeney GL, Aletti G, Podratz KC (2003). Endometrial cancer: predictors of peritoneal failure. *Gynecologic Oncology*.

[19] Hoekstra AV, Kim RJ, Small W (2009). FIGO stage IIIC endometrial carcinoma: prognostic factors and outcomes. *Gynecologic Oncology*.

[20] Havrilesky LJ, Cragun JM, Calingaert B (2005). Resection of lymph node metastases influences survival in stage IIIC endometrial cancer. *Gynecologic Oncology*.

[21] Lutman CV, Havrilesky LJ, Cragun JM (2006). Pelvic lymph node count is an important prognostic variable for FIGO stage I and II endometrial carcinoma with high-risk histology. *Gynecologic Oncology*.

[22] Chan JK, Cheung MK, Huh WK (2006). Therapeutic role of lymph node resection in endometrioid corpus cancer: a study of 12,333 patients. *Cancer*.

[23] Chan JK, Wu H, Cheung MK, Shin JY, Osann K, Kapp DS (2007). The outcomes of 27,063 women with unstaged endometrioid uterine cancer. *Gynecologic Oncology*.

[24] Neubauer NL, Havrilesky LJ, Calingaert B (2009). The role of lymphadenectomy in the management of preoperative grade 1 endometrial carcinoma. *Gynecologic Oncology*.

[25] Kilgore LC, Partridge EE, Alvarez RD (1995). Adenocarcinoma of the endometrium: survival comparisons of patients with and without pelvic node sampling. *Gynecologic Oncology*.

[26] Todo Y, Kato H, Kaneuchi M, Watari H, Takeda M, Sakuragi N (2010). Survival effect of para-aortic lymphadenectomy in endometrial cancer (SEPAL study): a retrospective cohort analysis. *The Lancet*.

[27] Ben-Shachar I, Pavelka J, Cohn DE (2005). Surgical staging for patients presenting with grade 1 endometrial carcinoma. *Obstetrics and Gynecology*.

[28] Selvaggi L, Loizzi V, Lorusso M, Demitri P, Cormio G (2010). Lymphadenectomy versus no lymphadenectomy in endometrial carcinoma: a retrospective analysis of 410 patients. *Journal of Gynecologic Surgery*.

[29] Bernardini MQ, May T, Khalifa MA (2009). Evaluation of two management strategies for preoperative grade 1 endometrial cancer. *Obstetrics and Gynecology*.

[30] Kitchener H, Swart AM, Qian Q (2009). Efficacy of systematic pelvic lymphadenectomy in endometrial cancer (MRC ASTEC trial): a randomized study. *The Lancet*.

[31] Panici PB, Basile S, Maneschi F (2008). Systematic pelvic lymphadenectomy vs no lymphadenectomy in early-stage endometrial carcinoma: randomized clinical trial. *Journal of the National Cancer Institute*.

[32] Orr JW, Holloway RW, Orr PF, Holimon JL (1991). Surgical staging of uterine cancer: an analysis of perioperative morbidity. *Gynecologic Oncology*.

[33] Todo Y, Yamamoto R, Minobe S (2010). Risk factors for postoperative lower-extremity lymphedema in endometrial cancer survivors who had treatment including lymphadenectomy. *Gynecologic Oncology*.

[34] Kong TW, Lee KM, Cheong JY (2010). Comparison of laparoscopic vs. conventional open surgical staging procedure for endometrial cancer. *Journal of Gynecologic Oncology*.

[35] Tozzi R, Malur S, Koehler C, Schneider A (2005). Analysis of morbidity in patients with endometrial cancer: is there a commitment to offer laparoscopy?. *Gynecologic Oncology*.

[36] Tozzi R, Malur S, Koehler C, Schneider A (2005). Laparoscopy versus laparotomy in endometrial cancer: first analysis of survival of a randomized prospective study. *Journal of Minimally Invasive Gynecology*.

[37] Walker JL, Piedmonte MR, Spirtos NM (2009). Laparoscopy compared with laparotomy for comprehensive surgical staging of uterine cancer: Gynecologic Oncology Group Study LAP2. *Journal of Clinical Oncology*.

[38] Mariani A, Dowdy SC, Cliby WA (2008). Prospective assessment of lymphatic dissemination in endometrial cancer: a paradigm shift in surgical staging. *Gynecologic Oncology*.

[39] Lee JY, Jung DC, Park SH (2010). Preoperative prediction model of lymph node metastasis in endometrial cancer. *International Journal of Gynecological Cancer*.

[40] Ballester M, Dubernard G, Rouzier R, Barranger E, Darai E (2008). Use of the sentinel node procedure to stage endometrial cancer. *Annals of Surgical Oncology*.

[41] Khoury-Collado F, Glaser GE, Zivanovic O (2009). Improving sentinel lymph node detection rates in endometrial cancer: how many cases are needed?. *Gynecologic Oncology*.

[42] Mais V, Peiretti M, Gargiulo T, Parodo G, Cirronis MG, Melis GB (2010). Intraoperative sentinel lymph node detection by vital dye through laparoscopy or laparotomy in early endometrial cancer. *Journal of Surgical Oncology*.

[43] Engelsen IB, Akslen LA, Salvesen HB (2009). Biologic markers in endometrial cancer treatment. *Acta Pathologica, Microbiologica et Immunologica Scandinavica*.

[44] Pradhan M, Abeler VM, Danielsen HE, Tropé CG, Risberg BÅ (2006). Image cytometry DNA ploidy correlates with histological subtypes in endometrial carcinomas. *Modern Pathology*.

[45] Trovik J, Wik E, Stefansson IM (2011). Stathmin overexpression identifies high-risk patients and lymph node metastasis in endometrial cancer. *Clinical Cancer Research*.

